# Editorial: Neurocognitive and Translational Science of Binge Eating: Understanding Mechanisms of Change

**DOI:** 10.3389/fpsyg.2022.904103

**Published:** 2022-04-25

**Authors:** Jose Carlos Appolinario, Allan Kaplan, Phillipa Jane Hay

**Affiliations:** ^1^Obesity and Eating Disorders Group, Institute of Psychiatry, Federal University of Rio de Janeiro, Rio de Janeiro, Brazil; ^2^Center for Addiction and Mental Health, Faculty of Medicine at the University of Toronto, Toronto, ON, Canada; ^3^School of Medicine, Translational Health Research Institute, Western Sydney University, Sydney, NSW, Australia

**Keywords:** binge eating, physiopathology, neuropsychology, neurophysiology, neuroimaging

Binge eating was initially described in 1959 by Albert Stunkard as an eating pattern associate with obesity (Stunkard, 1959). The major components of a binge are eating an objectively large amount of food and a feeling of loss of control over eating. However, growing evidence suggest that binge eating may also be associated with the ingestion of smaller amounts of foods (Palavras et al., 2013). Currently, episodes of binge eating are incorporated as diagnostic criterion of eating disorders in DSM-5 (American Psychiatric Association, 2013) and ICD-11(World Health Organisation, 2018). But recurrent binge eating episodes (RBE) in people without full-fledged criteria for an ED have clinical significance and are considered an increasing problem in the general population (Munsch et al., 2007; Appolinario et al., 2022).

Although binge eating itself is a well-established pattern of disordered eating behavior which impacts on both mental and physical wellbeing there is a need to better understand its nature in terms of varying degrees of dysfunction in psychological/biological systems. In line with this perspective, a series of advances in genetics, neuropsychology, neurophysiology, neuroimaging, and other biological markers have recently brought some light to the mechanisms underlying binge eating behavior (Kessler et al., 2016; Kober and Boswell, 2018). Some processes/domains have been associated with binge eating: (1) emotional reactivity/dysregulation, (2) a greater cognitive attentional bias toward food, (3) a decreased reward sensitivity, altered brain activation in regions associated with impulsivity and compulsivity. These discoveries are influencing current developments in the field, and we are now facing a move to translate these findings into new forms of intervention. This current Research Topic about neurocognitive and translational science of binge eating aims to compile studies from a multidisciplinary perspective to advance our knowledge of binge eating determinants and effective treatments.

Four studies included in the current issue explored binge eating determinants.

Seo and Lee investigated how shape and weight concerns (SWC) influence binge eating behavior based on attentional patterns toward high-calorie food cues. Although it is not a diagnostic criterion for the diagnosis, a group of individuals with BED showed high levels of overconcern about weight and shape (Ojserkis et al., 2012). The authors demonstrated that the level of SWC makes a unique contribution to binge eating initial orienting bias toward, and difficulty disengaging from, high-calorie food cues. This study of attentional bias elucidated the role of SWC as a potential maintenance factor of being concerned and binging in binge eating.

There is increasing evidence suggesting that higher levels of impulsivity may play a major role in overeating (Schag et al., 2013). Saruco and Pledger explored the role of impulsivity in overeating in a systematic review of brain inhibitory networks in individuals with high BMI and the role of BED in this association. The selected studies revealed significant BMI-dependent alterations of neural circuits primarily involving the frontal and limbic regions during cognitive inhibition and the presence of binge eating disorder results in further aggravation of those neural alterations. These results add to the evidence suggesting the involvement of neural circuits in eating and weight disorders and the possible role of binge eating in this mechanism.

Hiluy et al. conducted a systematic review investigating electrophysiological findings in binge-purge eating disorders (BP-ED). The authors presented their findings according to the EEG technique used. Studies with event-related potentials (ERP) in BP-ED using eating disorder-related and non-related stimuli suggested significant differences in N200, P200, P300, and LPP components in BP-ED participants compared to controls, indicating that this population exhibits impairments in selective attention, attentional allocation/processing, and allocation of motivational or emotion-based attention. In addition, EEG studies using frequency analysis reporting significant differences in beta activity in fronto-temporal and occipito-temporo-parietal areas in BP-ED individuals compared to controls, revealing a dysfunctional brain network. These electrophysiological findings are in line with current perspectives in the area and the authors suggest the potential utility of EEG/ERP as a potential “window” to better understand the pathophysiology of binge eating spectrum conditions.

Individuals with binge eating spectrum conditions usually have episodes of overeating with high energy/palatable (Bohon and Stice, 2012). In addition, those individuals when exposed to certain food cues demonstrated an impairment in brain circuits related to the reward system and executive function (planning, etc) (Kessler et al., 2016). Donnelly et al. (accepted) with neuroimaging investigated the neural responses to both low-energy and high-energy food images in three emotive categories (disgust; fear; happy) in BN and BED. The results in the low energy food condition indicate that binge frequency may be related to increased aberrant neural response. The neural response seen in the binge eating spectrum to disgust food images may indicate disengagement with these stimuli. In the high energy food condition, results demonstrate that neural activity in BN and BED patients decreases in response to high energy foods, suggesting disengagement with foods that may be more consistent with those consumed during a binge eating episode.

Three studies in this Research Topic investigated interventions based on these binge eating determinants.

Emotional reactivity, depression and anxiety have important roles in the development and maintenance of binge eating (Leehr et al., 2015). This interface was explored by Segura-Garcia et al. in a study aimed at evaluating the efficacy of vortioxetine (VTX) on depressive symptoms and in executive functions, eating behavior and body weight in patients with BED. Although preliminary, these authors found that VTX had a positive impact in depression, working memory, binge eating frequency/severity, night eating, food addiction, and body weight. They concluded that VTX can be a valid therapeutic option for patients with BED with comorbid depression for controlling the depressive symptoms, working memory, and eating behavior. Indeed, by acting on affective symptoms, neurocognitive functioning, and eating behaviors, it confirms the results already obtained with VTX in other disorders, expanding them to BED.

Lisdexamfetamine dimesylate (LDX) was the first medication approved for the treatment of BED by FDA in 2015 based on a robust development program (Appolinario et al., 2019). Although this agent demonstrated effectiveness in reducing binge eating frequency, its mechanism of action remains unknown. Griffiths et al. investigated the role of impulsivity in LDX-related reductions of binge eating (BE) episodes. There were significant associations between the degree to which subjective loss of control overeating, non-planning impulsivity and BE frequency reduced after 8 weeks of LDX. These data suggest that specific subjective measures of impulsivity may be able to predict who will have the greatest benefit from LDX treatment, and that reductions in BE frequency may be moderated by concurrent reductions in non-planning impulsivity.

Abnormalities in inhibitory control and attentional bias toward food were investigated by Ince et al. The authors assessed the underlying neuropsychological mechanisms of an inhibitory control training combined with anodal transcranial direct current stimulation (tDCS) of the right dorsolateral prefrontal cortex (DLPFC) by measuring certain ERPs components of patients with BED while performing a food antisaccade task. This study provides preliminary evidence for differing response inhibition processes among patients with BED when confronted with food through findings of less pronounced N200 and more pronounced ERN and P300 amplitudes in erroneous vs. correct saccades. As it predicts task performance on follow up assessments, P300 might be a potential marker for food related inhibitory control processes in BED.

In summary, the studies included in this current Research Topic: *Neurocognitive and Translational Science of Binge Eating: Understanding Mechanisms of Change* increase the knowledge about the nature of binge eating. We anticipate they will inform new perspectives for the treatment of this disordered eating behavior.

## Author Contributions

All authors listed have made a substantial, direct, and intellectual contribution to the work and approved it for publication.

## Conflict of Interest

The authors declare that the research was conducted in the absence of any commercial or financial relationships that could be construed as a potential conflict of interest.

## Publisher's Note

All claims expressed in this article are solely those of the authors and do not necessarily represent those of their affiliated organizations, or those of the publisher, the editors and the reviewers. Any product that may be evaluated in this article, or claim that may be made by its manufacturer, is not guaranteed or endorsed by the publisher.
